# Approach to the increase of depression and its treatment. Role of primary care. An opinion article

**DOI:** 10.3389/frhs.2025.1715754

**Published:** 2025-11-10

**Authors:** R. Cobos-Campos, S. Villullas, S. García de Andoin, I. Pérez, C. Bermúdez-Ampudia, E. López de Abechuco

**Affiliations:** 1 Primary Care, Epidemiology and Public Health Group, Bioaraba Health Research Institute, Vitoria-Gasteiz, Spain; 2 Research Network on Chronicity, Primary Care, Prevention and Health Promotion, Spain; 3 Associated Clinical Group, Bioaraba Health Research Institute, Vitoria-Gasteiz, Spain; 4 Osakidetza Basque Health Service, Vitoria-Gasteiz, Spain

**Keywords:** primary care, depression, COVID-19 disease, burnout professional, health care economics and organizations

Depression is one of the most prevalent psychiatric illnesses worldwide (1). The National Survey on Drug Use and Health revealed an unprecedented need for and use of mental health care resources. It is estimated that 5% of the world's adult population (1) suffers from depression with a prevalence of 4% among men and 6% among women (2). In Spain, 5.3% of the population over 15 years of age reported having depression in 2020, with a notable gender disparity: 7.2% of women compared to 3.2% of men (3–5).

The COVID-19 pandemic has had a profound impact on the mental health of the population. In 2020, approximately 21 million adults experienced a major depressive episode, and one in five Americans reported some form of mental illness during the pandemic period (6).

Moreover, according to a recent World Health Organization (WHO) report, both depression and anxiety increased by more than 25% in the first year of the pandemic (3, 7), especially in the 16–24 age group (2.7%). This rise has been driven, in part, by the context of social isolation experienced (4). Notably, the increase has also been more pronounced among women (0.6%) and in rural areas (0.7%) (4).

Depression is a common mental illness that can affect anyone. It is characterized by low mood or loss of interest in activities over extended periods (8). In addition, mental disorders contribute to 4.32% (9) of the global burden of disease, ranking them as the world's leading cause of disability and significantly contributing to workplace absenteeism (9–11).

Mental disorders often lead to a higher incidence of physical impairment (1) and greater likelihood of premature death compared to general population's expected age-related death rates (11). In addition, people with depression, regardless of age, are at increased risk of certain physical diseases (cardiovascular disease, stroke, diabetes, pain and Alzheimer's disease). The underlying reasons remain unclear. It may be attributed to reduced access to proper medical care, but there may also be physiological changes that impact on physical health (increased inflammation levels, changes in heart rate control and blood circulation, abnormalities in stress hormones production, metabolic changes…) (12).

Mental illnesses contribute significantly to morbidity, mortality and reduced quality of life worldwide (13, 14). Depression and anxiety, two common mental illnesses, were ranked respectively as the second and seventh leading causes of disability worldwide.

This complex relationship between mental and physical health highlights the need for developing strategies addressing both dimensions. In this regard, primary care is essential to tackle this growing public health concern.

Generally, primary care physicians treat mental symptoms as part of something, part of a larger, more general problem. The nature of primary care, is integrative. The more pronounced the physical symptomatology, regardless of whether the symptoms have a physical explanation or not, the greater the likelihood that a primary care patient has a mental health diagnosis (15). In other words, mental symptoms and disorders are concentrated precisely in those patients who visit their primary care physician for other reasons, such as physical illnesses or, at the very least, biomedical problems. Conversely, patients with psychological distress experience increased physical symptomatology (15).

The World Health Organisation (WHO) has published a new policy paper focusing on how countries can make mental health care more accessible and less stigmatizing through primary health care (16). The paper, entitled “Scaling up mental health services within the primary care approach: lessons from the WHO European Region”. Between one third and one half of all people presenting to primary care services do so because of mental health complaints, making primary care a crucial point for building trust in health systems. However, traditionally in many countries, in family health team models, if detected mental disorders, people may be immediately referred to specialist mental health services, where they often face long waiting times. These long delays can cause conditions to worsen, and the use of specialist services can also carry stigma, further discouraging people from seeking the help they need (16).

WHO refers primary care as the setting of choice to firstly address mental health problems. The accessibility and integrated approach of primary care make this first level of healthcare ideal to respond to the majority of mental health care needs, even in developed countries (17, 18).

Twenty five percent of individuals attending a health center have a diagnosable mental health condition, being depression the most predominant disorder. In fact, depression is the third most common cause for primary care consultation (17, 18). Therefore, it is necessary to implement collaborative strategies among different health care professionals, reinforcing the role of primary care in mental health care, and only referring to specialized services those patients requiring more intensive follow up (17–20). The risk of self-injury, the potential to harm others, or the presence of psychotic symptoms are critical factors when deciding the need for specialized psychiatric care. Geographical factors and available infrastructure also play a key role in assessing needs, since general practitioners are reluctant to refer a patient to specialized care if long journeys to hospital, often by public transport, are required. Moreover, insufficient cooperation between the primary care physician and patient is another important issue determining referrals to specialized care (21).

Collaborative care models are multicomponent interventions based on teamwork in order to redesign care delivery. Increased accessibility to psychological interventions by integrating clinical psychologists into the primary care team reduces medicalization of mild and/or moderate mental pathologies (20, 21).

The care manager is a key figure in collaborative care models. This role is assigned to primary care nurses, who, in close collaboration with primary care physician, are responsible not only for monitoring clinical progress and treatment adherence, but also for providing emotional and self-management support (17, 18, 22). This is a novel approach in our healthcare system, and implies profound changes in the way depression has traditionally been managed. Physicians have to relinquish responsibilities and nurse care managers have to assume new ones. Moreover, an additional challenge lies in establishing smooth and effective coordination among healthcare providers (17, 18). Such programs have demonstrated to be successful in previous experiences (17, 18); however, the approach needs to be bottom-up, involving front-line professionals in both, the development and implementation phases.

In spite of the high evidence of the need to implement these type of integrative programs, it is necessary to identify and to understand the factors and barriers, which make it difficult. Among them, we can mention the knowledge and skills of health care providers, the motivation for the change, the efficacy of health care centers to manage mental health disorders, the budget assigned to the mental health management and the credibility and confidence in the integrative models (23).

On the other hand, mental illness also has a huge economic impact. This impact has been estimated to reach up to 16.3 trillion dollars between 2011 and 2030 worldwide (24). In Spain, depression alone accounts a total cost of 6 billion euros. In particular, 67% of this amount is derived from productivity losses due to premature death and sick leave. It is therefore a major public health concern, but also a significant social and economic challenge (24).

**Figure 1:**
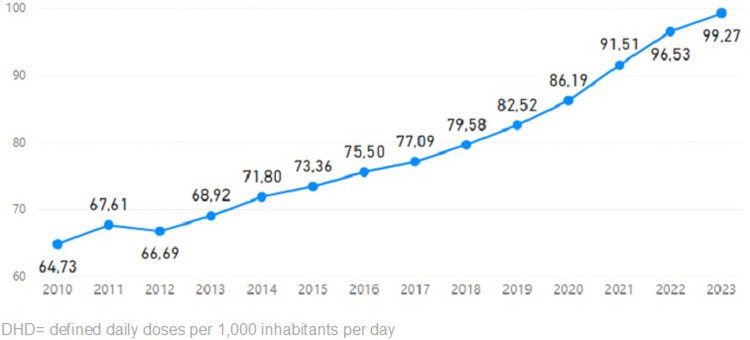
Variation of DHD of antidepressants in Spain over the years. Source: Spanish agency for medicine and health products.

The increase in depression diagnoses has been accompanied by a rise in the prescription of pharmacological treatment. Between 2015 and 2021, there has been an average increase of 15.1 DHD (defined daily doses per 1,000 inhabitants per day) (dayamong OECD countries (25). In Spain, this increase was even more pronounced, reaching up to 19.6 DHD (17). Figure 1 shows the upward trend in the number of DHDs in Spain over the years (26).

Nevertheless, recent studies suggest that an integrated strategy combining pharmacotherapy with psychological support is more effective than each treatment separately (27). The combined approach shows enhanced efficacy [Risk Ratio (RR) vs. psychotherapy RR = 1.25; 95% CI 1.09–1.43; vs. drugs RR = 1.27; 95% CI 1.12–1.43], compared to psychotherapy or pharmacotherapy individually (26). In fact, when treating depression, apart from pharmacotherapy and psychotherapy, stress management, social and community support, stigma and discrimination, and concomitant comorbidity treatments (28) should also be addressed.

In general, antidepressants, either alone or in a combined approach, should be started at a subtherapeutic dose, in order to assess patient tolerability, and then, gradually increased to a minimally effective dose. Moreover, it is important to highlight that antidepressants can cause side effects such as hyponatremia [OR = 3.160 (95%CI 1.911–5.225)] (29), as well as an increased risk of suicide [OR = 1.66 (1.37–2.02)] (30). Therefore, clinicians should ensure that these drugs are only prescribed to patients with a clear indication. Particular caution should be taken when prescribing antidepressants to patients with an increased underlying risk of seizures (30). In these cases, patients should be referred to mental health professionals.

Mental illness is one of the greatest challenges facing the healthcare system. Given its prevalence, on the rise after the COVID-19 pandemic, this problem is remarkably concerning among adolescents, since one in seven young people aged 10–19 years suffers from some mental disorder (19). Due to the enormous impact on health, and on personal, family, social and economic consequences, depression is a major social health issue that must be addressed by all the society, extending beyond the healthcare sector alone.

As mentioned above, the World Health Organisation (WHO) has long advocated for the integration of mental health services into primary care and community settings to address the large gap in mental health treatment (13). Commissioners and providers of mental health services for people with depression should ensure the effective delivery of treatments. This should build on the key functions of a catchment area-based community mental health service and be provided in the context of a coordinated primary and secondary care mental health service. It is necessary to support the integrated delivery of services across primary and secondary care, to ensure individuals do not fall into gaps in service provision (31, 32).

Integration of mental health services is seen as a feasible strategy to reach a large proportion of the population, reduce mental health stigma and address both mental and physical health outcomes (20).

Multiple studies suggest that integrated primary care-based programs are effective in improving mental health outcomes and quality of life in low- and middle-income countries (21, 33).

Despite their effectiveness, integrated programs have not yet been widely adopted, implemented and scaled up (34). First, there is a need to better understand what core program components and implementation strategies work. Second, the lack of experimental evidence needs to be addressed through pragmatic implementation-effectiveness trials in routine primary care and community settings.
